# Research and application of gas therapy in preventing biofilm associated infections

**DOI:** 10.4103/mgr.MEDGASRES-D-25-00075

**Published:** 2026-01-06

**Authors:** Pan Yang, Yadi Wang, Xueling Li, Junhong Lü

**Affiliations:** 1 College of Pharmacy, Binzhou Medical University, Yantai, Shandong Province, China; 2 Jinan Microecological Biomedicine Shandong Laboratory, Jinan, Shandong Province, China; 3 College of Public Health, Shanghai University of Medicine & Health Sciences, Shanghai, China; 4 Key Laboratory of Molecular Pharmacology and Drug Evaluation, Ministry of Education, School of Pharmacy, Yantai University, Yantai, Shandong Province, China

**Keywords:** anti-biofilm, biosynthesis, gas signaling, gas therapy, infections, metabolism, micro/nanobubbles, microbial biofilms, photodynamic therapy, quorum sensing

## Abstract

Conventional antibiotic therapies often fail to eradicate biofilms, which can lead to persistent infections and significant clinical challenges. Gas therapy, which utilizes the unique properties of gas molecules such as nitric oxide, carbon monoxide, hydrogen, and hydrogen sulfide, is emerging as a promising and innovative strategy to address these challenges. This review first highlights gas signaling in bacterial biofilms. It then goes on to list four types of gas therapy in detail: photothermal-enhanced gas therapy, photodynamic-activated gas therapy, micro/nanobubble-mediated gas therapy, and gas-based synergistic therapy. Their potential applications and future directions are also fully discussed. Due to its unique bioactivity, low resistance, and synergy with existing treatments, gas therapy has demonstrated significant potential in the prevention and treatment of biofilm-associated infections. However, overcoming delivery challenges, validating efficacy in large-scale trials, and developing standardized protocols are essential for its clinical translation. Future efforts should prioritize the integration of nanotechnology and mechanistic studies to unlock broader therapeutic utility.

## Introduction

Biofilm-related infections pose a significant global health challenge.^1^ Biofilms are complex microbial communities embedded within an extracellular polymeric substance (EPS) matrix that they secrete. This growth pattern provides enhanced resistance to antibiotics and the host’s immune responses. It is estimated that approximately 80% of bacterial infections in humans are associated with biofilms.^2^ Consequently, there is an urgent need for novel therapeutic strategies to address these infections.

**Figure 1:**
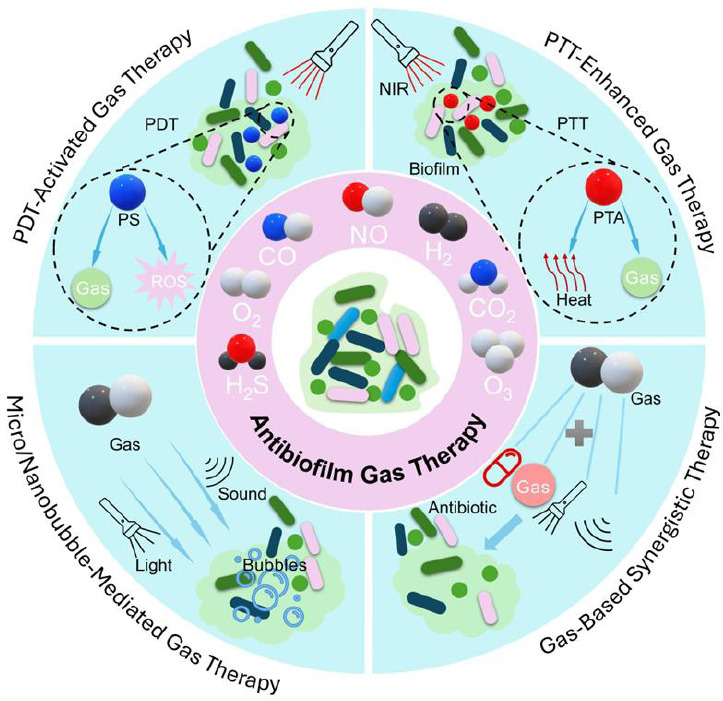
Gas therapy-based strategies for anti-biofilm applications. PTT-enhanced gas therapy: PTT uses light-responsive PTAs to enable the targeted release of gases under near-infrared irradiation. PDT-activated gas therapy: PDT combines PS, gas donors, and light to generate ROS. Micro/nanobubble-mediated gas therapy: Gas-filled microbubbles (1–10 µm) or nanobubbles (100–1000 nm) release gas in a precise, controlled manner. Gas-based synergistic therapy: The complementary mechanisms of multiple gases or gas-drug combinations are used to maximize therapeutic efficacy and minimize off-target effects. Created with Microsoft PowerPoint. CO: Carbon monoxide; CO_2_: carbon dioxide; H_2_: hydrogen; H_2_S: hydrogen sulfide; NIR: near-infrared; NO: nitric oxide; O_2_: oxygen; O_3_: ozone; PDT: photodynamic therapy; PS: photosensitizers; PTA: photothermal agent; PTT: photothermal therapy; ROS: reactive oxygen species.

Gas therapy has recently attracted widespread interest as an anti-biofilm strategy.^3^,^4^,^5^,^6^ In addition to the potent antimicrobial properties at low concentrations of gas molecules (nitric oxide (NO), carbon monoxide (CO), and hydrogen sulfide (H_2_S), among others), gas therapy offers unique advantages in combating biofilm-associated infections. Firstly, small gas molecules can more easily penetrate and diffuse deeply into the biofilms. Second, gases can simultaneously destabilize biofilms by inducing oxidative stress, metabolic partitioning and signaling inhibition, thereby amplifying oxidative stress and overcoming hypoxia-induced chemoresistance. Thirdly, gases have low cytotoxicity at controlled doses. Finally, gas therapy can be combined with antibiotics or other therapies to enhance efficacy. This review aims to provide an overview of recent advances in gas therapy for anti-biofilm applications, focusing on gas signaling, therapeutic strategies, and future directions (**Figure 1**).

## Search Strategy

To capture all relevant evidence, systematic searches were conducted across electronic databases, such as PubMed/MEDLINE, and Web of Science. Core keywords (e.g., “gas therapy,” “nitric oxide” OR “NO” OR “hydrogen sulfide” OR “H_2_S,” “nanobubble” AND “microbubble”) were identified and used for comprehensive retrieval, with non-biofilm studies exclusion.

## Gas Signaling in Microbial Biofilms

The formation and maintenance of biofilms are orchestrated by a trio of gas signaling molecules, particularly NO,^7^ CO,^8^ and H_2_S.^9^ These gaseous mediators integrate bacterial metabolism, quorum sensing (QS), and stress responses into a unified regulatory network that governs the initiation, maturation, and dispersal of biofilm.^10^

### Biosynthesis and metabolism of gas signaling molecules

Bacteria in biofilms use enzymatic pathways to synthesize and metabolize gas signaling molecules. The synthesis-degradation equilibrium is crucial to the structural integrity of biofilms. NO is primarily synthesized by bacterial nitrate reductase and nitrite reductase, playing a key role in regulating bacterial metabolism.^11^ CO, a by-product of hemoglobin degradation, modulates bacterial respiratory chains and energy metabolism by binding to metalloproteins.^8^,^12^ H_2_S, which is produced by enzymes such as cystathionine β-synthase and cystathionine γ-lyase, has a crucial role in maintaining redox balance and stress resistance.^10^,^13^

### Role of gas signaling during biofilm development

**Figure 2:**
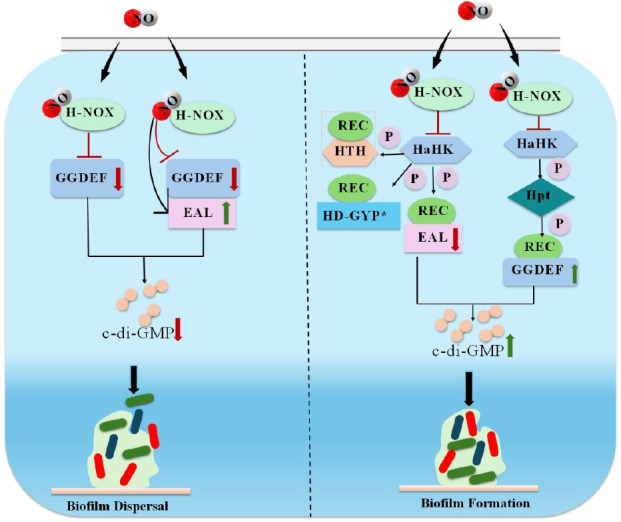
Dual impact of NO on bacterial biofilms. NO significantly alters bacterial biofilm formation by modulating c-di-GMP levels through two main pathways. Left: NO binds to H-NOX proteins, which then interact with catalytic CMEs (HaCMEs). This interaction inhibits the DGC activity in HaCMEs with only a GGDEF domain. In HaCMEs with both an active or inactive GGDEF domain and an EAL domain, H-NOX binding either maintains or reduces and/or activates PDE activity. This collectively decreases c-di-GMP, leading to biofilm dispersal. Right: NO binding to H-NOX affects its interaction with HaHK, thereby inhibiting HaHK autophosphorylation. This blocks phosphotransfer to response regulators like HnoC or directly to HaCMEs that fuse REC and GGDEF/EAL domains. The altered phosphorylation state of these HaCMEs inhibits PDE activity or activates DGC, increasing c-di-GMP and promoting biofilm formation. Reprinted from Yu et al.^14^ c-di-GMP: Cyclic di-guanosine monophosphate; CME: c-di-GMP metabolic enzyme; DGC: diguanylate cyclase; EAL: Glu-Ala-Leu motif; GGDEF: Gly-Gly-Asp/Glu-Glu-Phe motif; HaCME: H-NOX-associated c-di-GMP metabolic enzyme; HaHK: histidine kinase; HD-GYP: His-Asp and Gly-Tyr-Pro motif; H-NOX: heme-nitric oxide/oxygen; Hpt: histidine phosphotransfer protein; HTH: helix-turn-helix; NO: nitric oxide; PDE: phosphodiesterase.

Gas molecules exert stage-dependent control over biofilm development. For example, during the initial stage of biofilm formation, low concentrations of NO stimulate the production of cyclic diguanylate to increase the expression of bacterial surface adhesins.^7^ High concentrations of NO, on the other hand, induce bacterial stress responses and promote biofilm dispersal during biofilm maturation (**Figure 2**).^14^ In contrast, CO disrupts bacterial communication and impairs biofilm structure and maturation in a concentration-dependent manner. H_2_S affects biofilm stability by regulating bacterial energy metabolism and EPS production throughout the life cycle.

### Gas signaling crosstalk with quorum sensing

The gas signaling pathway and QS systems are closely linked.^15^ This crosstalk allows gas molecules to act as environmental switches. Under nutrient stress, NO affects the production of QS signaling molecules by regulating the activity of LuxR-type transcription factors, thereby promoting bacterial dispersal. Under oxidative stress, H_2_S modulates the redox state of QS systems.^16^

## Gas Therapy-Based Strategies for Anti-Biofilm Applications

### Photothermal therapy-enhanced gas therapy

Photothermal therapy (PTT) uses photothermal agents, including either nanomaterials or small molecules encapsulated within nanoparticles, to absorb near-infrared (NIR) light (700–950 nm) and convert it into localized heat. This allows for deep tissue penetration and the eradication of microbes by inducing bacterial membrane rupture and protein denaturation.^17^ To maximize therapeutic efficacy and safety, the controlled delivery of photothermal agents is essential. PTT can be integrated with light-stimulated gas-releasing agents to utilize light-responsive photothermal agents for synchronized gas release and photothermal effects.^18^ The synergistic effects of combing gas therapy and PTT offer several advantages for treating biofilms, including broad-spectrum antibacterial activity, excellent controllability, short treatment durations, and a low risk of bacterial resistance.^19^

**Figure 3:**
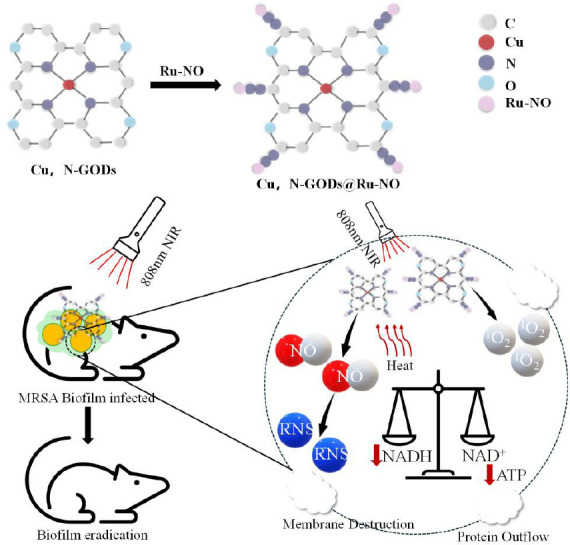
Photothermal therapy-enhanced gas therapy based on Cu, N-GQDs@Ru-NO nanozyme. The Cu, N-GQDs@Ru-NO nanozyme was developed by combining Cu, N-GQDs with a NO donor (Ru-NO). When exposed to 808 nm NIR light, this nanozyme acts like NADH dehydrogenase, oxidizing NADH to NAD^+^ and disrupting the bacterial redox balance. It also releases NO at the site of infection, eroding biofilms while promoting wound healing. Reprinted from Wang et al.^20^ Copyright 2023 Wiley‐VCH GmbH. ATP: Adenosine triphosphate; Cu, N-GQD: Cu, N-doped graphene quantum dot; MRSA: methicillin-resistant *Staphylococcus aureus*; NAD^+^: nicotinamide adenine dinucleotide; NADH: nicotinamide adenine dinucleotide reduced; NIR: near-infrared; NO: nitric oxide; RNS: reactive nitrogen species; Ru-NO: ruthenium nitrosyl complex, a NO donor.

Recent studies have explored the therapeutic potential of PTT-stimulated NO generation systems. For example, Wang et al.^20^ developed a nanozyme platform (Cu, N-GQDs@Ru-NO (Cu, N-doped graphene quantum dots are covalently functionalized with a NO donor, ruthenium nitrosyl)) which exhibits dual-modal antibacterial activity upon exposure to 808 nm NIR light (**Figure 3**).^21^ This system can convert photo-oxidizes nicotinamide adenine dinucleotide into NAD^+^ and simultaneously achieve NIR-triggered NO release. This synergistic mechanism effectively eradicates methicillin-resistant *Staphylococcus aureus* (*S. aureus*) biofilms. Polydopamine derivatives are another promising class of gas-releasing matrix for PTT applications. Lv et al.^21^ developed an intelligent antibacterial system (B@MPDA-Mal) by functionalizing polydopamine with maltotriose-decorated mesoporous polydopamine (MPDA-Mal) and incorporating the diazeniumdiolate NO donor BNN6. Yuan et al.^22^ further advanced this strategy by engineering deoxyribonuclease-CO@mesoporous polydopamine nanoparticles (DNase-CO@MPDA) nanoparticles with triple-functional design. These examples demonstrate the clinical potential of PTT-based gas therapy in effectively eliminating methicillin-resistant *S. aureus* biofilm infections and alleviating inflammation in treated wounds.

### Photodynamic therapy-activated gas therapy

Photodynamic therapy (PDT) is a minimally invasive, spatiotemporally selective approach with few side effects and a low risk of resistance.^23^,^24^ PDT uses photosensitizers, oxygen, and light to generate reactive oxygen species (ROS), which can damage biofilm components.^25^ However, the biofilm matrix barrier and hypoxic microenvironment can limit the efficacy of PDT. To address this issue, PDT-activated gas-releasing platforms have been developed to achieve synergistic effects. Lv et al.^26^ designed a phototherapeutic nanoplatform by loading the NO donor sodium nitroprusside (SNP) onto amino-modified mesoporous silica nanoparticles (MSNs), which were then modified with a porphyrin-based metal-organic framework (Ti-TCPP MOF), forming MSN@MOF/SNP NPs (MMS NPs). Under laser irradiation, these NPs release NO, thereby enhancing biofilm permeability and inducing ROS production. Similarly, Liu et al.^27^ developed a nanoplatform (ALCe6 NPs) by loading chlorin e6 (Ce6) onto nanoliposomes composed of L-arginine and soybean phospholipid-modified cholesterol. Under NIR light, Ce6 generates ROS, enabling controlled NO release. Both platforms demonstrate synergistic effects of ROS and NO in dispersing biofilms and eradicating drug-resistant bacteria. Cheng et al.^28^ created micellar nanoparticles by co-assembling diblock copolymers containing NO-releasing moieties and palladium-based photocatalysts. These nanoparticles adapt to the heterogeneous pH and oxygen conditions of biofilm microenvironments, releasing NO under red light irradiation. They were found to effectively eradicate ciprofloxacin-resistant *Pseudomonas aeruginosa* biofilms *in vitro* and in a skin infection mouse model.^29^ Ma et al.^30^ also designed a PDT-driven CO-releasing system (Ce6&CO@FADP) for eliminating *S. aureus* and *Escherichia coli* (*E. coli*) biofilms. Under 665 nm NIR light, Ce6&CO@FADP generates O_2_ and H_2_O_2_, with the latter oxidizing metal-based CO-releasing molecules to release CO. The combination of O_2_ and CO works together to eradicate biofilms *in vitro* and alleviate subcutaneous bacterial infections *in vivo*.

### Micro/nanobubble-mediated gas therapy

Ultrafine bubbles, including microbubbles and nanobubbles (diameters < 1 μm), interact effectively with biological cells and biofilm structures. These bubbles detach biofilms and prevent their formation through capillary forces at the air-liquid interface. Furthermore, the free radicals generated by ultrafine bubbles can oxidize cell walls and interfere with bacterial division.^31^,^32^,^33^,^34^ Studies show that ultrafine bubbles containing air, CO_2_, or N_2_, when combined with antimicrobial solutions, can significantly enhance the eradication of *E. coli* biofilms on various surfaces.^35^,^36^ However, their limited stability restricts penetration into certain treatment sites.^37^ Oxygen nanobubbles can inhibit or promote microbial growth depending on the type of biofilm.^38^,^39^,^40^,^41^,^42^,^43^ Ye et al.^44^ developed a combined therapy using oxygen-loaded lipid nanobubbles, silver nanoparticles, and PDT. The oxygen nanobubbles overcame biofilm hypoxia, thereby enhancing PDT performance and promoting the oxidative dissolution of silver. This effectively inhibited microbial growth in a mouse model. Ozone ultrafine bubble water inhibits *Candida albicans* biofilm formation by suppressing hyphal growth and inducing oxidative stress responses to promote wound healing.^45^ Laser-induced vapor nanobubbles enhance antibiotic penetration into biofilms by generating pressure waves that disrupt their structures. Teirlinck et al.^46^ demonstrated that vapor nanobubbles significantly improve the efficacy of antimicrobial agents against *Pseudomonas aeruginosa* and *S. aureus* biofilms. Hematoporphyrin monomethyl ether combined with microbubbles improves ROS production and cavitation intensity, effectively inhibiting *E. coli* biofilms.^47^,^48^

### Gas-based synergistic therapy

Combining gas therapy with other treatments enhances efficacy and reduces side effects. Tang et al.^49^ developed NIR-stimulated NO-releasing gold nanocubes to eradicate methicillin-resistant *S. aureus* biofilms. Similarly, Klinger-Strobel et al.^50^ embedded Mn_2_(CO)_10_ into electrospun fibers for CO release under 405 nm light, reducing bacterial counts in biofilms by 70%. Li et al.^51^ engineered an ultrasound-responsive tricarbonyl rhenium(I) complex (RePy-TPE) for dual CO gas therapy and sonodynamic therapy. Under ultrasound, RePy-TPE generates ROS and releases CO, effectively eradicating *Mycobacterium tuberculosis* and *E. coli* biofilms.^52^ Zhou et al.^53^ synthesized biofilm-responsive polymeric CO-releasing micelles that, combined with subminimal inhibitory concentrations of antibiotics, exhibit potent activity against multidrug-resistant pathogens. Jiang et al.^54^ developed a multifunctional nanoplatform integrating anti-biofilm, anti-inflammatory, and osteogenic capabilities. Under NIR irradiation, the platform releases NO and CO, generating ROS for biofilm elimination and tissue repair. Yuan et al.^55^ designed an L-arginine-functionalized mesoporous polydopamine nanoplatform loaded with indocyanine green. NIR irradiation induces ROS production and NO release, enabling synergistic biofilm ablation.

To evaluate the unique advantages and limitations of current biofilm-targeting modalities and guide their clinical application, we provide a comparative overview of PTT, PDT, microbubble-, and nanobubble-based gas therapies. This comparison highlights critical parameters including mechanisms of action, penetration efficacy, therapeutic specificity, and suitability for distinct clinical scenarios (**Table 1**).

**Table 1:** Comparative analysis of photothermal therapy, photodynamic therapy, microbubble, and nanobubble-based gas therapies

Parameter	Photothermal therapy	Photodynamic therapy	Microbubble-based therapy	Nanobubble-based therapy
Mechanism	Used photothermal agents (e.g., gold nanoparticles) to convert light to heat, killing cells	Used photosensitizers activated by light to generate reactive oxygen species, killing cells	Gas-filled microbubbles (1-10 μm) collapse under ultrasound, enhancing drug/gas delivery	Gas-filled nanobubbles (100-1000 nm) release therapeutic gases (e.g., O_2_, NO) via stimuli
Activation method	Near-infrared light	Visible or near-infrared light	Ultrasound	Ultrasound, pH, or enzymatic triggers
Primary target	Cancer cells, infected tissues	Cancer, biofilms, microbial infections	Vascularized tumors, drug delivery, biofilm disruption	Hypoxic tumors, biofilm infections, targeted drug delivery
Penetration depth	High (near-infrared penetrates 2-3 cm)	Low-moderate (limited by light penetration, ~0.5-1 cm)	Moderate (dependent on ultrasound focus)	High (nanoscale size enables tissue extravasation)
Efficacy	High for localized tumors (> 80% in preclinical models)	High in superficial infections/biofilms (70-90%)	Enhanced drug/gas delivery by 2-5 times; biofilm disruption (~60-70%)	Improved gas/drug bioavailability; biofilm disruption (~70-80%)
Key advantages	Deep tissue penetration, minimal invasiveness	High specificity, dual antimicrobial and anti-biofilm action	Real-time imaging compatibility (ultrasound contrast), localized delivery	Prolonged circulation, enhanced tissue penetration, controlled gas release
Key limitations	Risk of overheating healthy tissue, requires precise targeting	Limited penetration depth, photosensitizer toxicity	Short half-life, limited to vascularized areas	Complex synthesis, potential immune clearance
Clinical applications	Solid tumor ablation, bacterial infection treatment	Skin cancers, chronic wound infections, dental biofilms	Ultrasound-guided drug delivery, thrombolysis, biofilm-associated infections	Tumor hypoxia alleviation, antimicrobial gas delivery, biofilm eradication

NO: Nitric oxide; O_2_: oxygen.

## Potential Applications of Gas Therapy

### Overcoming biofilm resistance and enhancing antibiotic efficacy

The structural organization of biofilms with spatial heterogeneity and chemical gradients leads not only to an EPS matrix barrier for limiting antibiotic penetration, but also to hypoxia microenvironments induced chemoresistance. Due to their small molecular size and high diffusivity, gas molecules have a promising ability to overcome these resistance mechanisms.^56^ For instance, NO can disrupt the EPS matrix and induce bacterial stress responses, thereby increasing membrane permeability and promoting antibiotic uptake.^57^ In addition to targeting biofilms directly, gas therapy can also enhance the efficacy of antibiotics by altering the metabolic activity and gene expression of bacteria embedded in biofilm. For example, H_2_S affect bacterial metabolic states, rendering biofilms more susceptible to the effects of antibiotics.^5^ A preclinical study has demonstrated the synergistic effects of combining gas therapy with antibiotics, with the combination of NO donors with ciprofloxacin significantly improving the eradication of *Pseudomonas aeruginosa* biofilms.^58^

### Application prospects in preventing biofilm formation

Gas therapy also shows great potential for preventing biofilm formation via various mechanisms.^59^ For example, NO inhibits bacterial attachment and aggregation, thereby preventing the initial development of biofilms.^60^ CO affects biofilm formation and stability by suppressing EPS production and interbacterial communication.^61^ H_2_S inhibits the early maturation of biofilm by modulating bacterial metabolism.^62^

### Potential in treating medical device-related infections

Medical device-related infections pose significant clinical challenges.^63^,^64^ Gas therapy provides innovative solutions for the treatment of such infections. Incorporation gas-releasing systems into medical devices enables sustained and controlled therapeutic delivery. For instance, NO-releasing coatings on urinary catheters significantly reduce the incidence of catheter-associated urinary tract infections.^65^ Similarly, CO-releasing materials on orthopedic implants lower the risk of implant-related infections.^61^ These applications highlight the prophylactic potential of gas therapy in clinical settings.

## Challenges and Future Directions

Gas therapy demonstrates distinct advantages over standard treatments and emerging antibiofilm strategies owing to its unique mechanisms and critical functional parameters (**Table 2**). Unlike standard antibiotics, which exhibit limited efficacy against biofilms due to poor penetration of the EPS barrier, gaseous molecules bypass this resistance via their small molecular size and high diffusivity.^5^ While antibiotics typically target single metabolic pathways (e.g., cell wall synthesis), gas therapy employs multimodal mechanisms.^5^,^66^ Compared with species-specific emerging strategies like phage therapy, gas molecules exhibit broad-spectrum activity against polymicrobial biofilms–a critical advantage given the clinical prevalence of complex, multispecies communities on medical devices and chronic wounds. These resilient consortia derive resistance from structural heterogeneity, cross-species metabolic cooperation, and EPS-mediated protection. Gas therapy counteracts these mechanisms through multi-target physicochemical actions.^67^,^68^ Compared with PDT and sonodynamic therapy, gas therapy overcomes limitations such as PDT inefficiency induced by hypoxia.^68^ Unlike enzyme-based approaches (e.g., DNase), which only degrade specific matrix components, gas therapy achieves synergistic biofilm eradication by disrupting the matrix and inhibiting bacterial metabolism.^5^ Despite significant progress in gas therapy, key challenges remain. Future research should focus on:

**Table 2:** Comparison of gas therapy with standard treatments and emerging antibiofilm strategies

Therapy	Mechanism	Biofilm penetration	Resistance development	Specificity	Application	Safety	Key finding/limitation
Gas therapy (e.g., NO)	Disrupted biofilm matrix via signaling pathways	High (gas diffusion)	Low	Moderate	Inhalation/topical	Moderate (dose-dependent toxicity)	Effective biofilm dispersal; requires controlled dosing. Potential toxicity at high doses.
Standard antibiotics	Inhibited bacterial cell processes (e.g., cell wall synthesis)	Low (blocked by matrix)	High	Broad-spectrum	Oral/IV/topical	Moderate (side effects)	Poor penetration into biofilms; high resistance rates. Often combined with debridement.
Phage therapy	Bacteriophages lyse specific bacterial cells	Moderate	Low-Moderate	High (strain-specific)	Topical/systemic	High	Strain-specific efficacy; resistance possible but phages evolve. Limited to known pathogens.
Quorum sensing inhibitors	Blocked bacterial communication signals	Moderate	Low	Broad	Topical/oral	High	Prevented biofilm formation; limited efficacy against mature biofilms.
Nanoparticles (e.g., Ag)	Released ions disrupting bacterial membranes	High (nanoscale size)	Low	Broad-spectrum	Topical/coatings	Low (toxicity at high doses)	Broad activity but cytotoxicity concerns. Often used in medical device coatings.
Enzyme-based (e.g., DNase)	Degraded biofilm matrix components (e.g., eDNA)	Moderate	Low	Moderate	Topical	Moderate (immune reactions)	Synergistic with antibiotics; limited standalone efficacy.
Photodynamic therapy	Light-activated ROS generation kills bacteria	Low (light-dependent)	Low	Moderate	Topical (light-accessible)	Moderate (ROS damage)	High efficacy in accessible areas; limited by light penetration depth.

Ag: Silver; DNase: deoxyribonuclease; eDNA: environmental DNA; IV: intravenous injection; ROS: reactive oxygen species.

(a) Development of advanced gas delivery systems: while current gas delivery approaches are limited by factors such as slow or uncontrolled release, next-generation systems should integrate smart stimuli-responsive mechanisms. These systems could use external triggers, such as near infrared light or ultrasound, or biomimetic signals (e.g., bacterial secretions) to enable targeted, on-demand drug release.^67^

(b) Elucidation of mechanistic pathways: While the anti-biofilm mechanisms of NO (e.g., nitrosative stress, cyclic diguanylate modulation) and CO (e.g., ROS generation) are partially understood, the role of other gases, such as H_2_S, remain unclear. Further research is needed to define the optimal therapeutic doses and mechanisms of action for different gases across various biofilm types.^69^

(c) Expansion of pathogen targets: Current gas therapy strategies focus primarily on biofilms of *S. aureus* and *Pseudomonas aeruginosa*. Future studies should explore applications against Gram-negative bacteria, mycobacteria, and fungal biofilms to enable personalized treatment regimens for diverse infections.^51^

(d) Exploration of combination therapies: Combining gas therapy with conventional antibiotics, PDT, or PTT has shown improved efficacy. Future research should explore novel combinations to reduce antibiotic use and combat the rise of multidrug-resistant “superbugs.”^67^

(e) Biosafety and clinical translation: While gases are generally biocompatible at therapeutic concentrations, gas carriers such as metal-based CO-releasing molecules may pose toxicity risks. Systematic assessment of cell and blood compatibility, biodistribution, metabolism, and long-term biosafety is essential for clinical adoption.^70^

(f) Development of gas-carrying nanobubbles: Nanobubbles exhibit excellent biocompatibility and enhance gas delivery and biofilm disruption. However, research in this area is still in its nascent. Future efforts should focus on optimizing gas-nanobubble combinations to address different biofilm infections.^71^,^72^

## Conclusion

This review design has its limitations, which must be acknowledged. Firstly, our analysis was mainly based on *in vitro* and preclinical animal studies, which reflects the early stage of clinical translation for most gas-based therapies. This could restrict their direct application in the clinical setting. Secondly, significant heterogeneity existed across the included studies about experimental models (e.g. bacterial strains and biofilm maturation stages), gas delivery systems and efficacy metrics. This complicated quantitative cross-comparisons of therapeutic outcomes. Thirdly, excluding non-English publications and relying on major databases may have resulted in the omission of relevant preclinical data or regional innovations. Lastly, the rapidly evolving nature of advanced delivery systems meant that our synthesis could not encompass very recent technological breakthroughs. Taken together, these factors limit the generalizability of the translational potential, but also highlight critical knowledge gaps for future research.

In summary, gas therapy represents a transformative approach to addressing biofilm-related infections and overcoming antibiotic resistance. Although it is still in its early stages, gas therapy aligns with the urgent need for innovative biofilm management strategies. With continued advancements in delivery systems, mechanistic understanding, and combination therapies, gas therapy is poised to become a cornerstone in the prevention and treatment of biofilm-associated infections, paving the way for its clinical translation and widespread application.

## Data Availability

*All data generated or analyzed during this study can be obtained from the corresponding author.*
